# The Dopaminergic Energy Crisis of Adolescent Depression: Mitochondrial Bioenergetics Control of Nucleus Accumbens Signaling

**DOI:** 10.1007/s12264-026-01658-7

**Published:** 2026-06-23

**Authors:** Salvatore Nesci

**Affiliations:** Department of Veterinary Medical Sciences, University of Bologna, 40064 Bologna, Ozzano Emilia Italy

Depression emerging in adolescence is not simply adult major depressive disorder at an earlier age. It arises during a developmental interval in which reward circuitry is being recalibrated, motivational priorities are being reshaped, and mesocorticolimbic dopamine projections remain unusually plastic. Developmental work indicates that adolescence is marked by ongoing refinement of dopamine axon targeting, including Netrin-1/DCC-dependent organization of dopaminergic connectivity involving the nucleus accumbens (NAcc) [1]. In parallel, adolescent anhedonia has become increasingly recognized as a dimensional, treatment-relevant phenotype linked to disturbed reward processing rather than a secondary byproduct of low mood alone.

The NAcc is especially well-positioned to convert developmental vulnerability into psychiatric symptomatology. As the ventral striatal hub that integrates dopaminergic input from the ventral tegmental area with cortical, thalamic, amygdalar, and hippocampal information, it determines whether effort is mobilized, reward is anticipated, and motivational salience is assigned. Human and translational work increasingly places NAcc-centered dysfunction at the core of depression-related anhedonia [2]. However, the field still lacks an integrative model explaining why reward signaling fails so persistently in some young patients, particularly those who do not respond to standard first-line treatments. A compelling possibility is that the missing layer is bioenergetic. Mitochondria are not passive ATP generators; they are regulators of calcium buffering, vesicle cycling, reactive oxygen species (ROS), inflammatory signaling, and cell-type-specific plasticity [3]. In affective disorders broadly, mitochondrial dysfunction and inflammation appear bidirectionally linked [4]. In the NAcc specifically, manipulation of mitofusin-2 (MFN2), a core regulator of mitochondrial fusion and trafficking, is sufficient to alter depression- and anxiety-related behaviors, indicating that mitochondrial state in accumbal neurons is behaviorally causal rather than epiphenomenal. Yet this concept has not been developed mechanistically in adolescent depression, where anhedonia is arguably the most reward-specific symptom domain [5].

Disturbed mitochondrial energy metabolism in depression is unlikely to be strictly NAcc-specific; rather, it appears to involve a broader cortico-limbic-midbrain network, including the prefrontal cortex, hippocampus, and dopaminergic midbrain [6]. The NAcc remains a particularly compelling focus because it integrates ventral tegmental area dopaminergic input with cortical and limbic afferents that regulate reward, motivation, reinforcement learning, and stress responsivity. Its relevance is therefore not limited to reward processing, but reflects its position as a convergence node where bioenergetic failure can directly impair motivational output. This is especially pertinent during adolescence, when mesolimbic dopamine circuitry remains developmentally plastic, and NAcc targeting is still being refined [1]. Moreover, mitochondrial regulation in the NAcc is mechanistically supported by evidence that MFN2 modulates depression- and anxiety-related behaviors.

## The Bioenergetic Hypothesis: From Oxidative Stress and Inflammation to Dopamine Hypofunction

The NAcc is likely a particularly vulnerable locus for this cascade because adolescence is not a quiescent period for mesolimbic circuitry. Developmental studies show that dopamine pathway organization and terminal targeting remain dynamic during adolescence [7]. At the same time, accumbal neurons are undergoing synaptic remodeling and functional specialization. Under these conditions, mitochondrial stress may have outsized consequences: not just reduced signaling in an established circuit, but maladaptive maturation of a circuit still under construction. That distinction is important. In adults, mitochondrial dysfunction may degrade an already configured reward system; in adolescents, it may bias the developmental set-point of reward sensitivity itself. This model also helps specify why the clinical phenotype is anhedonia rather than generic psychomotor slowing alone. NAcc dopamine does not merely encode pleasure; it calibrates incentive salience, willingness to expend effort, and learning from motivationally relevant outcomes. If phasic dopamine transients are flattened because terminals cannot meet energetic demand, reward prediction and action energization both weaken. The adolescent then reports not only reduced pleasure, but diminished wanting, reduced anticipatory engagement, and a subjective sense that effort is metabolically “not worth it.” That profile maps closely onto the contemporary multidimensional concept of anhedonia in youth [8].

The anhedonia in adolescent depression can be conceptualized as the behavioral output of a localized, developmentally amplified bioenergetic failure within NAcc reward circuitry. The sequence is as follows: stress and inflammatory activation increase cytokine signaling and oxidative burden; mitochondrial respiration becomes less efficient; ATP supply falls while ROS rise; mitochondrial quality control and organelle dynamics deteriorate; dopaminergic terminals and NAcc neurons then fail to sustain high-fidelity reward signaling. However, this framework is an interdependent mitochondrial stress network rather than a fixed linear sequence, consistent with evidence showing that inflammation in youth depression is present but heterogeneous, and that anhedonia in adolescents is specifically associated with inflammatory measures rather than uniformly across all symptom dimensions [9].

The first critical step is ATP insufficiency. In adolescent depression, impaired ATP supply is likely to reflect reduced mitochondrial oxidative phosphorylation efficiency, increased ROS burden, disturbed calcium handling, and defective mitochondrial quality control, all of which lower neuronal energy availability for synaptic transmission [6]. Although direct NAcc-specific evidence in adolescents remains limited, emerging adolescent data support the relevance of mitochondrial energy metabolism by showing ATP-related abnormalities that improve with treatment. Dopamine neurotransmission is energetically expensive. Action-potential propagation, calcium handling, vesicle docking, vesicular monoamine transport, endocytosis, and transporter recycling all depend directly or indirectly on mitochondrial ATP availability. Vesicular monoamine transporter 2 (VMAT2) is especially relevant because vesicular sequestration protects dopamine from cytosolic oxidation while also determining how much transmitter is available for release [10]. Dopamine transporter (DAT), in turn, strongly shapes extracellular dopamine dynamics and presynaptic homeostasis. Thus, VMAT2 and DAT are central determinants of release amplitude, reuptake kinetics, and terminal vulnerability. In a low-ATP state, vesicle acidification, vesicular filling, and terminal recovery after firing are all predicted to become less efficient, yielding smaller phasic dopamine signals in the NAcc [11].

The second step is oxidative stress. Dopamine is intrinsically oxidation-prone outside the vesicular lumen. When mitochondrial impairment reduces efficient sequestration or increases terminal stress, cytosolic dopamine becomes more vulnerable to oxidation, generating reactive intermediates that further damage proteins, lipids, and mitochondrial components [12]. This creates a feed-forward loop: impaired mitochondria increase ROS; ROS impair dopamine handling; poorly sequestered dopamine produces additional oxidative stress; and the terminal becomes progressively less capable of sustained signaling. ROS excess, mtDNA injury, and disturbed mitophagy are central outcomes of mitochondrial dysfunction, while the dopamine literature identifies VMAT2-dependent vesicular sequestration as a key defense against dopamine-mediated oxidative injury [13].

**Fig. 1:**
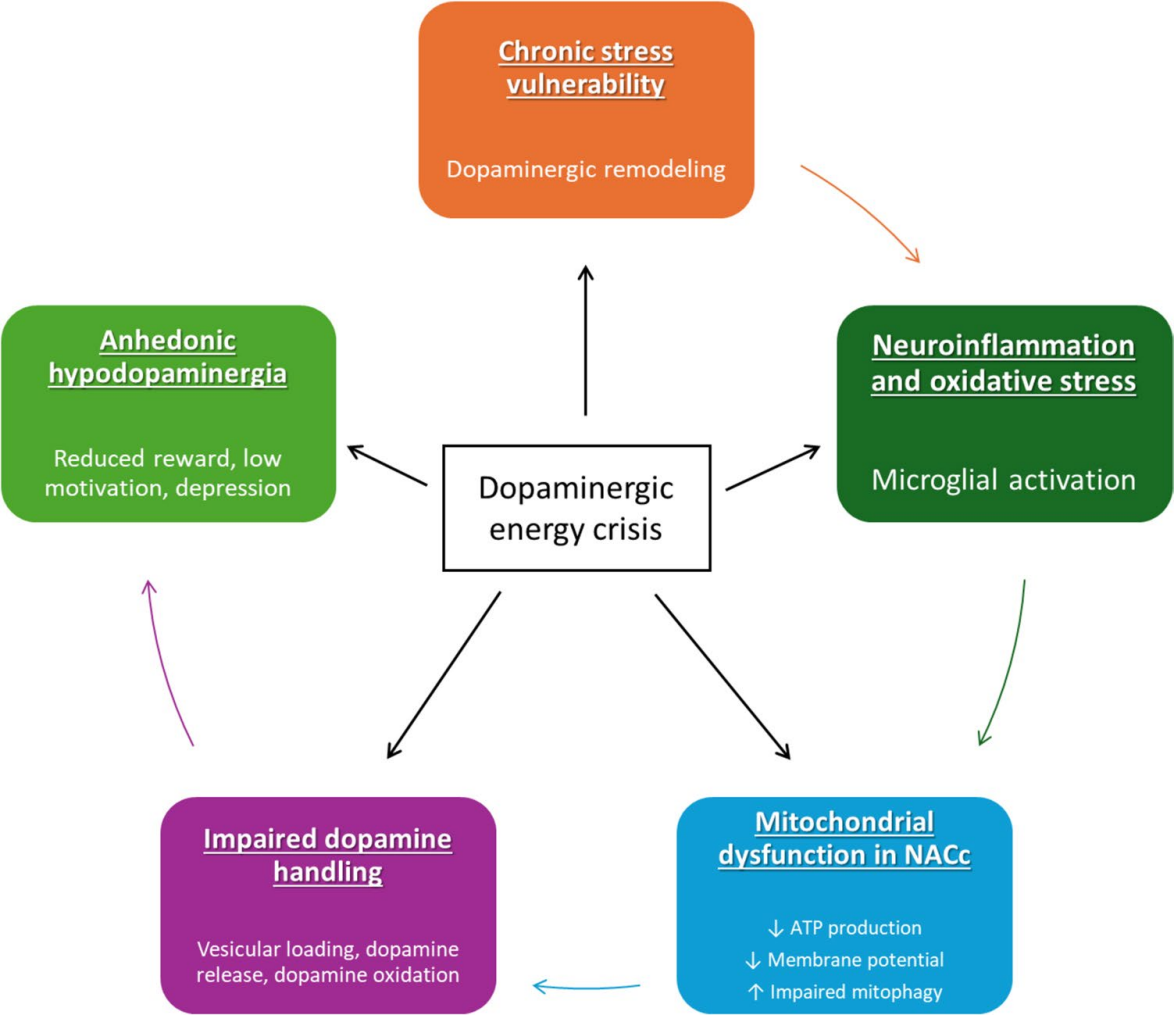
Unified cascade linking oxidative stress, inflammation, mitochondrial dysfunction, and reduced dopamine signaling in adolescent anhedonia. During adolescence, psychosocial stress and developmental vulnerability may promote inflammatory activation and oxidative stress within the nucleus accumbens (NAcc) reward circuitry. These processes impair mitochondrial ATP production, redox homeostasis, and quality control in accumbal neurons and dopaminergic terminals, leading to reduced vesicular dopamine loading, destabilized release, altered reuptake energetics, and increased cytosolic dopamine oxidation. The resulting hypodopaminergic state diminishes reward salience and motivational drive, contributing to the emergence of anhedonia in adolescent depression.

The third step is inflammatory amplification. Mitochondria and inflammation are tightly interlocked [3]. Damaged mitochondria can amplify inflammatory signaling through ROS and danger-associated molecular patterns, whereas cytokines can worsen mitochondrial function and redox imbalance (Figure 1). In depression, this reciprocity has already been recognized at the systems level. In youth, the literature suggests subtle but meaningful inflammatory dysregulation, and symptom-level approaches increasingly indicate that reward dysfunction and anhedonia may be especially relevant targets for immunobiological stratification [14]. Thus, rather than considering inflammation and reward dysfunction as separate explanatory modules, it may be more accurate to view inflammation as an upstream force that destabilizes mitochondrial competence in reward circuitry.

Importantly, current evidence does not yet prove that mitochondrial dysfunction within the juvenile NAcc is the singular initiating lesion in depression. The direct evidence remains incomplete. What the field does have is a convergent scaffold: adolescent reward circuitry is developmentally labile; NAcc mitochondrial regulation influences depression-related behavior; inflammation and mitochondrial dysfunction interact in affective disorders; and anhedonia in youth is closely tied to reward-circuit pathology. The hypothesis is therefore strong enough to organize experiments, even if it remains partially inferential at present [9].

The ATP deficit-oxidative stress-inflammation cascade reflects dysfunction of the neuron-glia metabolic unit rather than neurons alone. Astrocytes sustain energy balance, antioxidant defense, and neurotransmitter clearance; their impairment lowers the energetic reserve of NAcc circuits and weakens dopamine signaling. Microglia add an immunometabolic component by linking mitochondrial stress to cytokine release, ROS generation, and maladaptive synaptic remodeling processes, especially relevant to adolescent depression, where early inflammation alters microglial spine engulfment and induces depressive‑like phenotypes [15]. Moreover, sex-dependent mitochondrial biology warrants consideration. Estrogen regulates mitochondrial bioenergetics, and these interactions may partly contribute to sex-dependent vulnerability in depression [16].

## Therapeutic Implications of the Oxidative Stress-Inflammation-Mitochondrial Dysfunction Network in Adolescent Depression

Reduced dopamine production should be interpreted broadly. It includes diminished synthesis support under inflammatory stress, but more importantly, reduced effective dopamine availability at the synapse because newly synthesized dopamine is not efficiently packaged, protected, released, or recycled [10]. From a functional standpoint, a terminal with normal synthetic machinery but defective bioenergetics behaves like a hypodopaminergic terminal. This is one reason why purely transmitter-centric models can miss the true lesion: the molecule may be present, yet the circuit is underpowered [17]. This framing may also explain why serotonergic treatment alone is often insufficient for adolescents whose depression is dominated by anhedonia. If the principal bottleneck is not receptor occupancy but terminal energy failure and redox imbalance in the NAcc, then symptom persistence is expected. Indeed, clinical and translational work increasingly recognizes anhedonia as a marker of worse course and poorer treatment response, reinforcing the need for mechanism-based subtyping rather than syndromic uniformity (Fig. 1).

The immediate implication is therapeutic reframing. For non-responder youth with prominent anhedonia, the aim may need to shift from “increase monoamine tone” to “restore reward-circuit bioenergetic competence”. That does not eliminate standard psychotherapy or pharmacotherapy; it changes the biological target hierarchy. A mitochondria-centered approach would predict greater benefit from interventions that improve oxidative phosphorylation efficiency, mitochondrial quality control, redox buffering, and inflammatory tone, particularly when combined with interventions that re-engage reward learning behaviorally. Lifestyle interventions are especially attractive in adolescents because they are scalable and mechanistically pleiotropic. Exercise is the clearest candidate: across the mitochondrial literature, it promotes mitochondrial biogenesis, enhances antioxidant defenses, and improves quality control pathways involving AMPK and PGC-1α [18]. In mice, exercise supports the reward function of the ventral tegmental area-NAcc pathway by enhancing mitochondrial bioenergetics, normalizing dopaminergic signaling, and strengthening synaptic resilience, thereby helping restore motivation through improved oxidative metabolism in mesolimbic neurons. In a developmental psychiatry framework, exercise may therefore do more than improve mood nonspecifically. It may directly raise the energy ceiling of reward circuitry and counter ROS-driven degradation of dopaminergic signaling. Sleep stabilization and nutrition likely operate in the same general direction, though the adolescent depression evidence remains less mature mechanistically.

Pharmacologically, the field should think beyond classic antidepressant categories. Mitochondria-targeting or redox-modulating strategies as preliminary preclinical or early clinical signals include N-acetylcysteine-based approaches, CoQ_10_-related strategies, acetyl-L-carnitine combinations, and other metabolic adjuncts [19]. The evidence in youth remains limited and should not be overstated, but the therapeutic logic is strong: adolescents with inflammatory-anhedonic phenotypes may be the subgroup in whom mitochondrial interventions are most rationally deployed. A further implication is biomarker development. If the model is correct, non-responder youth with anhedonia should show some combination of inflammatory enrichment, altered peripheral redox markers, reward-circuit functional abnormalities, and signatures of impaired mitochondrial fitness. No single biomarker is likely to suffice. The appropriate framework is multimodal stratification: symptom dimension plus inflammation plus reward-circuit readout plus metabolic signature [9]. That is already the direction in which adolescent anhedonia research is moving.

## Future Directions

The field needs direct measurements of mitochondrial respiration, membrane potential, ROS load, calcium buffering, and mitophagy in adolescent NAcc microcircuits, ideally distinguishing dopaminergic terminals, interneurons, astrocytes, and microglia. The MFN2 in the NAcc demonstrates that mitochondrial dynamics are behaviorally consequential [20], but it does not yet resolve which cellular compartments are primary in adolescent anhedonia. A decisive next step would be developmental single-cell or spatial multi-omics combined with functional mitochondrial assays in stress-sensitive adolescent models. Mitophagy is a particularly strong candidate because it sits at the intersection of stress adaptation, ROS control, and synaptic endurance. The core experimental question is whether defective removal of damaged mitochondria in adolescent dopaminergic terminals or accumbal neurons precedes blunted reward behavior, or merely follows it. Longitudinal studies should manipulate PINK1/Parkin-linked pathways, fusion-fission balance, and mitochondrial trafficking during adolescence while measuring NAcc dopamine release, effort-based decision making, and anhedonia-like behavior. Adolescent depression is biologically heterogeneous. Mechanism-based trials should enrich for adolescents with prominent anhedonia, evidence of inflammatory activation, and reward-circuit impairment, then test whether mitochondrial-directed interventions normalize both symptoms and circuit function. This is the necessary translational bridge: not simply asking whether a metabolic adjunct reduces total depression scores [6], but whether it rescues the specific motivational computations most dependent on NAcc dopamine.

## Conclusion

The adolescent anhedonia may reflect a failure of reward circuitry to meet its energetic demands during a uniquely vulnerable developmental window. The NAcc is not just a reward node; it is a metabolically intensive interface where inflammation, stress, dopamine, and mitochondrial quality control converge. When that interface enters a bioenergetic crisis, phasic dopamine signaling loses amplitude and reliability, motivational salience collapses, and the adolescent experiences the world as less worth pursuing. That framework integrates oxidative stress, inflammation, mitochondrial dysfunction, and dopaminergic reduction into a single developmental pathophysiology. It also offers a more actionable direction for treatment-resistant youth depression: repair the energetics of reward.
